# A physiologically based kinetic model for elucidating the *in vivo* distribution of administered mesenchymal stem cells

**DOI:** 10.1038/srep22293

**Published:** 2016-02-29

**Authors:** Haolu Wang, Xiaowen Liang, Zhi Ping Xu, Darrell H. G. Crawford, Xin Liu, Michael S. Roberts

**Affiliations:** 1 Therapeutics Research Centre, School of Medicine, The University of Queensland, Princess Alexandra Hospital, Woolloongabba, QLD 4102, Australia; 2 Department of Biliary-Pancreatic Surgery, Ren Ji Hospital, School of Medicine, Shanghai Jiao Tong University, 1630 S. Dongfang Road, Shanghai, 200127, China; 3 Australian Institute for Bioengineering and Nanotechnology, The University of Queensland, St Lucia, QLD 4072, Australia; 4 School of Medicine, The University of Queensland, Gallipoli Medical Research Foundation, Greenslopes Private Hospital, Greenslopes, QLD 4120, Australia; 5 School of Pharmacy and Medical Science, University of South Australia, Adelaide, SA 5001, Australia

## Abstract

Although mesenchymal stem cells (MSCs) present a promising tool in cell therapy for the treatment of various diseases, the *in vivo* distribution of administered MSCs has still been poorly understood, which hampers the precise prediction and evaluation of their therapeutic efficacy. Here, we developed the first model to characterize the physiological kinetics of administered MSCs based on direct visualization of cell spatiotemporal disposition by intravital microscopy and assessment of cell quantity using flow cytometry. This physiologically based kinetic model was validated with multiple external datasets, indicating potential inter-route and inter-species predictive capability. Our results suggest that the targeting efficiency of MSCs is determined by the lung retention and interaction between MSCs and target organs, including cell arrest, depletion and release. By adapting specific parameters, this model can be easily applied to abnormal conditions or other types of circulating cells for designing treatment protocols and guiding future experiments.

Mesenchymal stem cells (MSCs), also called multipotent mesenchymal stromal cells, are self-renewing, nonhematopoietic somatic stem cells comparable to embryonic stem cells in terms of their multipotency and proliferative and differentiation potential. Due to their multilineage differentiation potential and immunomodulatory properties, MSCs present a promising tool in cell-based therapy for treatment of various nonhematopoietic diseases, such as myocardial infarction, liver cirrhosis, spinal cord injury, cartilage damage and diabetes^1^,^2^,^3^. After the first clinical trial employing MSCs to treat osteogenesis imperfecta published in 1999^4^, the number of registered clinical trials significantly increased, reaching 344 in 2013^5^. Restoring the viability and function of MSCs in anatomically complex organs (*e.g.* the liver, heart, and brain) remains a challenge for systematic MSC transplantation. Although functional improvements following the delivery of MSCs have been extensively explored in various diseases, our current understanding of the *in vivo* behavior and distribution of administered MSCs is limited, which seems to hamper further transition of MSC transplantation from experimental trials to standard clinical procedures. Previous studies showed that most of MSCs were entrapped in the lung immediately after intravenous injection, with some MSCs undergoing apoptosis^6^. After about 10 min, these trapped MSCs gradually returned to the blood circulation and redistributed to other organs^7^. Finally only a small fraction of MSCs were found to survive, migrate to and engraft in the target organs. Thus, it would be important to characterize the *in vivo* distribution of MSCs following intravascular administration to predict their survival and homing to target organs^6^.

A number of published model have the potential to characterize the *in vivo* behavior of administered stem cells. The long-term replication, differentiation, or apoptosis of stem cells could be predicted by stochastic model^8^,^9^ or time-variant clustering model^10^. A computational cell motility model has been developed to probe the migration mechanism of cells^11^. And the population dynamics of administered cells may be predicted using a recently developed mathematical model^12^. However, none of the above-mentioned published models could elucidate the concentration-time profiles of administered cells in organs. There is still a lack of a proper model to characterize the *in vivo* distribution of administered stem cells. It has been reported that the dynamics of systematically administered MSCs were similar to that of inert micrometer-scale particles injected into the bloodstream of animals^13^. Therefore the complex, yet regulated, *in vivo* kinetics of administered MSCs are amenable to pharmacokinetic model building and analysis. During the past 30 years, physiologically based kinetic (PBK) models have been successfully applied to analyze the kinetics of small molecules, antibodies, nanoparticles and lymphocytes^14^,^15^. Such model is based on the anatomical structure of the living systems, with each important organ regarded as an individual compartment. All compartments are connected by blood flow^14^. Compared to empirical kinetic models, PBK modeling has the potential for interspecies scaling, which allows prediction of compound pharmacokinetics in humans using animal data. By systematically examining the effects of changing individual model parameters, PBK models can identify key parameters and their values, and suggest possible strategies for improvements in biodistribution. Therefore, quantitatively analyzing the *in vivo* distribution of MSCs with PBK modeling has the potential to identify the barriers to MSCs delivery, and propose designs of new formulations and dosing regimens to maximize the therapeutic activity.

In this study, we developed a simple PBK model to characterize the *in vivo* kinetics of MSCs from biodistribution data of green fluorescent protein (GFP) expressed MSCs intravenously injected into mice. Being the first effort to model the distribution of administered stem cells, this model invoked assumptions based on direct visualization of MSCs in specific organs at the cellular level using high resolution multiphoton microscopy. The utility of the model was examined across species and administration routes by extrapolation of this model to rats and humans, as well as to intra-hepatic arterial injection. The clinical utility of the model was also tested with data obtained from stem cell-based therapies to patients with liver cirrhosis. This PBK model provides a general framework for the study of *in vivo* distribution of therapeutic cells to design treatment protocols and to guide future experiments.

## Results

### Disposition of MSCs at organ level

**Figure 1:**
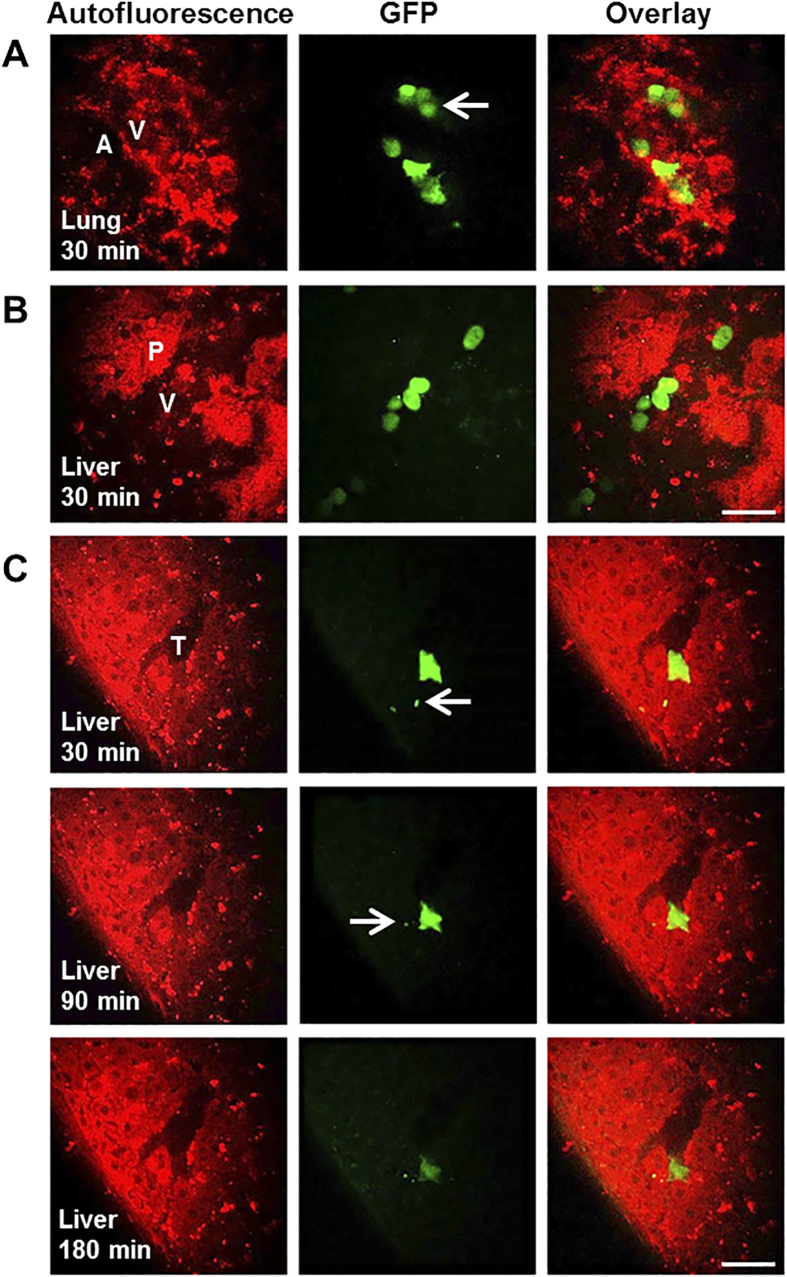
Disposition of MSCs at organ level. (**A**) At 30 minutes post injection, the MSCs were found entrapped in the microvessels of the lung while no cell was found in the surrounding collagens or alveoli in the lung. Some MSCs less than 10 μm in diameter and may pass through the capillaries. (**B**) At 30 minutes post injection, some MSCs in liver were found accumulated and moving in vessels with diameters greater than those of MSCs and no cell was found extravasate into the liver parenchyma. (**C**) The depletion of MSC in the liver after intravenous injection. After entrapped at the junction of the terminal portal venule and sinusoids at 30 minutes post-injection, one MSC slowly became fragmented with reduced fluorescence suggestive of depletion. No MSCs was observed to cross the vessel membrane to the liver parenchyma. Images were recorded at λ_Exc_/λ_Em_: 740/350 to 450 nm for the endogenous autofluorescence of the lung and liver (red, left column), and λ_Exc_/λ_Em_: 900/450 to 515 nm for fluorescence of GFP (green, middle column). The right column represents fused images. Scale bar: 40 μm, and the white arrow points towards the MSCs with smaller sizes. A, alveoli; V, vessels; P, parenchyma; T, terminal portal venule.

The spatiotemporal disposition of GFP-MSCs in organs at the cellular level was explored using multiphoton microscopy (MPM). Figure 1A,B shows representative images of MSCs distribution in lung and liver at 30 min after intravenous injection. The MSCs were quickly observed in the microvessels of the lung and liver, instead of extravascular migrating into the surrounding collagens or alveoli of the lung and parenchyma of the liver. The size of MSCs was determined to be 20.1 ± 1.2 μm in mouse blood using MPM (Supplementary Fig. S1), and confirmed by bright-field microscopy (22.0 ± 2.6 μm, Supplementary Fig. S2). Some MSCs became passively entrapped in small-diameter blood vessels, and some were found to accumulate and move in vessels with diameters greater than those of MSCs (Fig. 1B), suggesting the existence of both passive and active organ retention of MSCs. MSCs with smaller sizes (around 10 μm) were observed in organ capillaries (Fig. 1A), suggesting the possibility of MSC deformability which has been reported previously^16^. Few MSC was detected extravascular migrating up to 24 hours following intravenous injection. Figure 1C depicts the depletion process of one representative MSC in liver captured by real-time intravital imaging. After entrapped into the junction of the terminal portal venule and sinusoids (30 minutes post-injection), cell fragmentation was gradually observed with reduced fluorescence due to cell depletion.

### Development of PBK model

**Figure 2:**
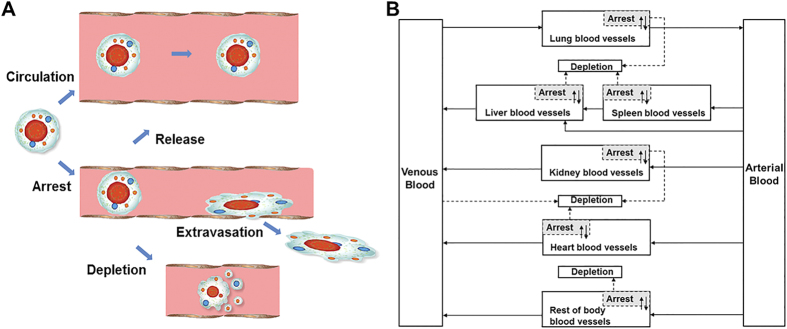
Hypothesis and schematic diagram of the PBK model for the *in vivo* fate of MSCs. (**A**) Assumptions for modeling based on direct visualization of MSCs in specific organs at the cellular level using high resolution multiphoton microscopy. After intravenous injection, MSCs were transported to blood vessels of organs via systemic circulation. After reaching the organs, some MSCs became entrapped in microvessels due to large sizes or adhered to the endothelial wall. These MSCs could be released back to blood circulation or eliminated after depletion. The process of tissue integration and differentiation of the arrested MSCs was much slower extending from 24 to 72 hours post-injection. (**B**) Schematic diagram of the PBK model for the *in vivo* fate of MSCs. Solid arrows indicate blood flow, dashed grey arrows indicate the depletion of MSCs and grey boxes indicate the arrested MSCs isolated from blood circulation as in the extravascular space of organ.

The PBK model of MSCs in mice was developed based on the above observations and on the published intravital microscopic details of administered MSCs^16^,^17^,^18^. After intravenous injection, MSCs were transported to blood vessels of organs by organ blood flow via the systemic circulation. This process is assumed to be very fast. As shown in Fig. 2A, after reaching the organs, some MSCs became entrapped in microvessels due to their large sizes or temporarily adhered to the endothelial wall. A portion of these entrapped MSCs could be released back to the blood circulation or eliminated after depletion. These arrest-release-depletion processes were assumed to follow first-order kinetics with rate constants of *k*_*arrest*_, *k*_*release*_, and *k*_*depletion*_, respectively. Tissue integration and differentiation of the arrested MSCs were not included in the model as these processes were much slower^19^,^20^ and had less impact on the MSC circulation and distribution at the organ level in the short term. All MSCs were assumed to act independently with no intercellular feedback loops or obligatory connections. For example, the entrapment of one MSC would not trigger the apoptosis or release of another. In summary, the *in vivo* kinetics of MSCs in this model was assumed to be governed by two processes: (1) transport to the organ via systematic circulation; (2) interaction with blood vessels of organs.

To build the PBK model, the whole body was separated into eight compartments: arterial blood, venous blood, lungs, spleen, liver, kidneys, heart and the rest of body. All compartments were interconnected via the systemic blood circulation (Fig. 2B). Key components included in the model were species-specific physiological parameters (body weight, organ volume and blood flow, given in Supplementary Table S1) and MSC-specific parameters (partition coefficient, arrest rate constant, release rate constant and depletion rate constant).

### Comparison of PBK model predictions with experimental data

**Figure 3:**
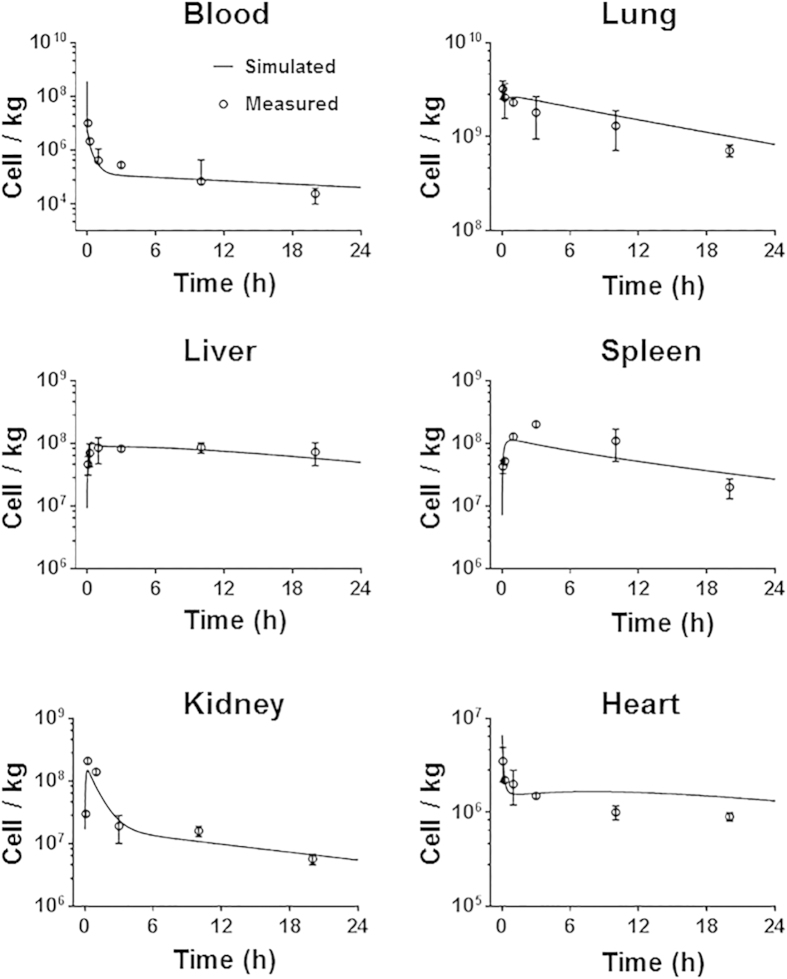
Model calibration results with experimental data. Mice were intravenously injected with 5 × 10^5^ MSCs (*n* = 5). The solid line in each panel represents the concentration-time profile of the MSCs simulated by the PBK model while the closed circles represent measured biodistribution data. Concentration of the MSCs is expressed as number of cells per kilogram of tissue. The data are expressed as mean ± s.d. The initial concentrations for organs (0 cell/L) are not shown because a base-10 log scale is used for the concentration.

After intravenous injection, the time profile of MSC levels in venous blood exhibited a two phase decay corresponding to a fast distribution and a relative slow elimination process. Lung, liver, spleen and kidney were major organs of MSC accumulation. MSCs displayed different patterns of concentration-time profiles in these organs. As shown in Fig. 3, the observed time profiles of MSCs concentration in mouse blood and organs were adequately described by the developed PBK model with an overall regression coefficient (*R*^2^) of 0.966 (Supplementary Fig. S3), indicating high goodness-of-fit of model calibration results. However, despite the adequate overall predictions, it should be noted that the model predicted a rapid decrease of MSC concentration in blood within 5 min after injection. There is a lack of experimental data at this early time point to confirm this, which requires further experiments to either validate this prediction or revise the model accordingly.

Table 1 summarized the MSC-specific parameters for each organ. The highest arrest rate constant was obtained for the lung estimated by curve fitting (5.434 h^−1^), indicating that MSCs are predominantly entrapped in the lung after *in vivo* administration. Blood showed the highest depletion rate constant (0.636 h^−1^), suggesting its role as major elimination organ. The depletion rate constant in kidney was found to be highest among all organ compartments (0.151 h^−1^), which was consistent with the results from whole-body imaging and radioactivity counting of urine after injection of ^99m^Tc labeled MSCs^21^. Our estimates suggest that about 28% of the transplanted MSCs survive *in vivo* 24 hours after intravenous injection. Similar survival rate have been obtained by intravital imaging of rat cremaster muscle microcirculation to track intraarterially delivered MSCs^16^.

To determine the effect of each parameter on the model simulation, a sensitivity analysis was performed. The relative sensitivity coefficient (RSC) for the concentration of MSCs in liver and heart are shown in Supplementary Fig. S4, because liver cirrhosis and myocardial infarction are two common diseases that have been treated with MSCs clinically. We selected 24 hours post-injection, when the amount of circulating MSCs decreased to a relatively steady state. The concentration of MSCs in the liver at 24 h post injection was highly sensitive to the depletion rate constant of liver, the release rate constant of lung, the partition coefficient and arrest rate constant of liver and lung. Similar effects of these parameters on the heart were observed. The depletion and arrest rate constant of heart, the arrest rate constant of lung, and the partition coefficient of heart and lung had a high impact on the concentration of MSCs in heart at 24 h post injection.

### Model evaluation with independent rodent data

**Figure 4:**
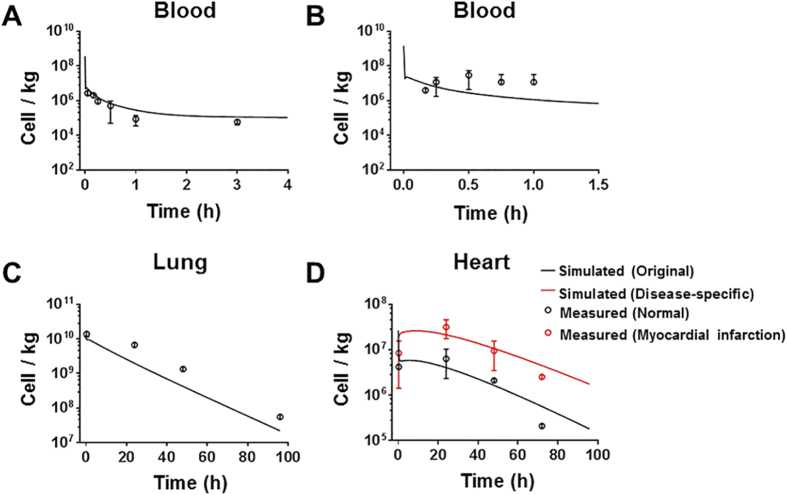
Model evaluation results with independent external datasets from mice. (**A**) Mice were intravenously injected with 5 × 10^5^ MSCs^22^ (*n* = 5). (**B–D**) Mice were intravenously injected with 2 × 10^6^ MSCs^7^ (*n* = 6). The solid line in each panel represents the concentration-time profile of the MSCs simulated by the PBK model while the closed circles represent measured biodistribution data. Concentration of the MSCs is expressed as number of cells per kilogram of tissue. The data are expressed as mean ± s.d. The initial concentrations for organs (0 cell/L) are not shown because a base-10 log scale is used for the concentration.

The validity of the PBK model was first evaluated with data from Shim *et al.*^22^ and Lee *et al.*^7^ where MSCs (5 × 10^5^ and 2 × 10^6^ cells) were intravenously administered to normal and diseased mice. All physiological parameters and MSC-specific parameters in the model were maintained constant. As shown in Fig. 4A,C,D, the model adequately predicted the MSC concentrations in blood in normal mice from the dataset of Shim *et al.*^22^, and MSC concentrations in lung and heart in normal mice from the dataset of Lee *et al.*^7^. However, as shown in Fig. 4B, the predicted MSC concentration in blood only slightly increased at about 3 min post-injection, while Lee *et al.* observed a much more substantial reappearance of MSCs in blood (2% to 3% of administered MSCs) after a lag period about 10 min^7^. A possible reason for this difference could be the different methods of MSC quantification, where the observed data were measured by quantitative assays for DNA of MSCs and our model was based on the data from flow cytometry analysis. Another reason is that mechanistic considerations, such as cell aggregation, changes in flow to organs, were not included in modeling, while these mechanisms might become more relevant to MSC distribution when higher concentration are administered. In the dataset from Shim *et al.*^22^, the distribution of MSCs did not differ significantly between normal and osteoarthritis-induced mice. However, the model underestimated the MSC concentration in the heart with myocardial infarction from the dataset of Lee *et al.*^7^ (Fig. 4D), indicating the disease effect on MSC distribution. We then recalibrated the model to data from mice with myocardial infarction^7^, to estimate the diseases-specific heart-related parameters (Supplementary Table S2). As shown in Fig. 4D, the MSC concentration in the infarcted hearts was more accurately predicted by the same PBK mode with re-estimated diseased-specific parameters than with original parameters (the comparison of precision is shown in Supplementary Table S3). The re-estimation results indicated that higher concentration of MSC is related to higher arrest and less depletion of MSC in heart in disease status.

**Figure 5:**
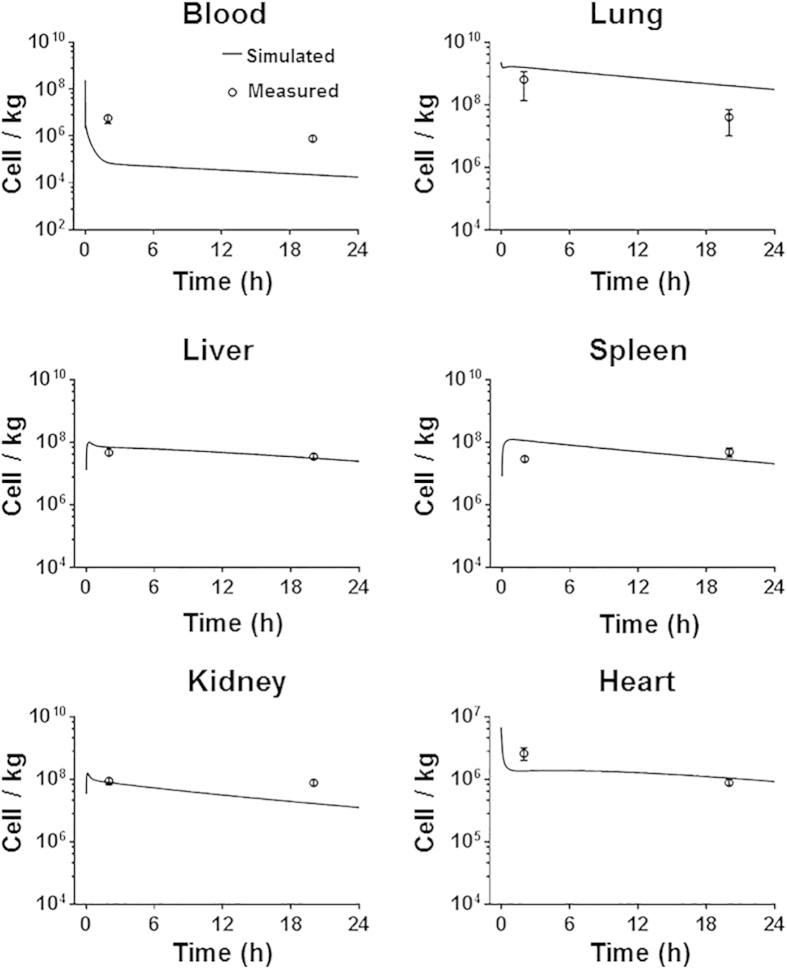
Model evaluation results with independent external datasets from rats. Rats were intravenously injected with 3.2 × 10^6^ MSCs^21^ (*n* = 9). The solid line in each panel represents the concentration-time profile of the MSCs simulated by the PBK model while the closed circles represent measured biodistribution data. Concentration of the MSCs is expressed as number of cells per kilogram of tissue. The data are expressed as mean ± s.d. The initial concentrations for organs (0 cell/L) are not shown because a base-10 log scale is used for the concentration.

To further evaluate the predictive applicability of the PBK model across species, simulations were compared with published experimental data for rat^21^. Physiological parameters of rats (given in Supplementary Table S1) were obtained from literature^23^,^24^. MSC-specific parameters in the model were maintained constant. As shown in Fig. 5, the MSC concentrations in the lung, liver, spleen, kidneys and heart were predicted adequately by the model, but the blood levels were underestimated. It should be noted that MSCs in that study were ^99m^Tc labeled and distribution was measured by nuclear imaging. Therefore, residual radioactivity of cell fragments in blood before excretion may result in overestimation of MSC concentration in nuclear imaging. In contrast, our model was based on the data from flow cytometry analysis, which largely reflected the concentration of live cells. Overall, there was a good correlation (*R*^2^ = 0.922) between PBK model estimates and independent rodent data (Supplementary Fig. S5), indicating that the model shown here is applicable to predict the *in vivo* distribution of administered MSCs across rodents. However, the time courses of this datasets have only two data points (2 hours and 20 hours) for each organ. More detailed data are needed to adequately evaluate the potential inter-species predictive capability of this model.

### Model predicting the *in vivo* distribution of therapeutic stem cells in humans

**Figure 6:**
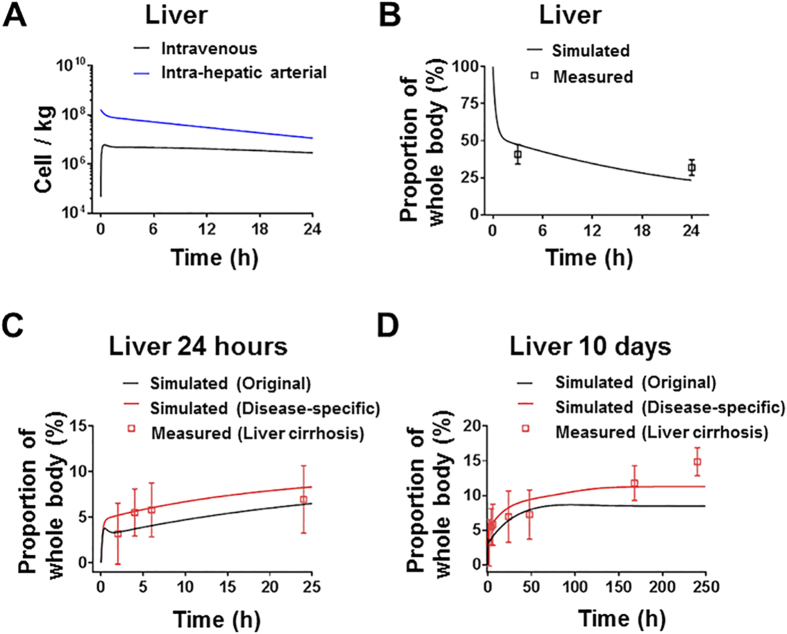
Model evaluation results with independent external datasets from humans. (**A**) The PBK model suggests that the time profiles of MSC concentration in liver significantly differ between patients after intravenous or intra-hepatic arterial injection of the same number (8.5 × 10^8^) of MSCs. (**B**) Patients were intra-hepatic arterially injected with an average of 8.5 × 10^8^ radiolabeled BMMCs^26^ (*n* = 8). (**C,D**) Patients were intra-hepatic arterially injected with an average of 3.2 × 10^8^ radiolabeled MSCs for 24 hours and 10 days, respectively^27^ (*n* = 4). The residual radioactivity in the liver is expressed as the proportion of the whole body radioactivity. The solid line in each panel represents the concentration-time profile of the cells simulated by the PBK model while the closed squares represent measured biodistribution data. The data are expressed as mean ± s.d.

This model was used to predict the *in vivo* distribution of the therapeutic stem cells in patients with liver cirrhosis after intravenous or intra-hepatic arterial injection. Physiological parameters of humans (given in Supplementary Table S1) were obtained from literature^23^,^24^,^25^, MSC-specific parameters in the model were maintained constant. As shown in Fig. 6A, our model suggests that the time profiles of MSC concentration in liver significantly differ between patients after intravenous or intra-hepatic arterial injection of the same number (8.5 × 10^8^) of MSCs. The model successfully predicted the proportion of bone marrow-derived mononuclear cells (BMMCs) in the liver to the whole body at 3 and 24 hours after injection for the data from Couto *et al.*^26^ (Fig. 6B). However, it underestimated the proportion of MSCs in the liver to the whole body after intra-hepatic arterial injection for the data from Gholamrezanezhad *et al.*^27^ (Fig. 6C). We then recalibrated the model to data from human with liver cirrhosis^27^, to estimate the diseases-specific liver-related parameters (Table S2). As shown in Fig. 6D, the MSC concentration in the cirrhotic liver was more accurately predicted by the same PBK mode with re-estimated diseased-specific parameters than with original parameters (the comparison of precision is shown in Supplementary Table S3). The re-estimation results indicated that higher concentration of MSC is related to higher partition and arrest of MSC in liver in disease status.

## Discussion

Although hundreds of studies have reported the cell biodistribution in the field of stem cell-based therapy, no integrating model characterizing the *in vivo* kinetics of these cells has been developed with respect to pharmacological effects and therapeutic thresholds. In the present study, we developed a model based on direct visualization of GFP-labeled MSCs disposition in the mice at the cellular level in specific organs. Importantly, the mouse cells used in this study were expanded to a similar size as the human MSCs currently used in clinical trials. Compared with the previously published PBK model of lymphocytes^15^, our model is especially applicable to circulating cells with large sizes (MSCs or cancer cells are 1.5 to 4 times larger than lymphocytes). This model is more useful for clinical applications since a less complicated framework and parameters were employed. In the future, the predictive power of this model is likely to improve with the incorporation of new parameter values or advanced microscopic details as they become available.

One of the advantages of PBK modeling over traditional empirical kinetic modeling is the ability to provide time profiles of cell concentration in individual organs. The *in vivo* distribution of MSCs characterized mathematically in the present study will better inform the dosing regimens of cell-based therapies. For example, it may be expected that higher administered numbers of MSCs should result in more MSC engraftment and better functional outcomes. However, in a rat model of brain injury, no additional enhancement of neurological function was observed after increasing the dose of intravenous injected MSCs by 3-fold^28^. Thus, simply increasing the number of delivered cells may not improve the overall outcome. The sensitivity analysis of our PBK model showed that the accumulation of MSCs in the lung adversely affected the delivery of therapeutic cells to other target organs, evident by the concentrations of MSCs in the target organs were sensitive to changes in the partition coefficient, arrest or release rate constant in the lung. The partition coefficient, arrest and depletion rate constant in target organs also had a high impact on the concentration of MSCs. Thus, instead of increasing the dose, possible strategies to further improve the target efficiency of cell-based therapies would be bypassing the initial lung entrapment and enhancing organ-specific capture by modulating cell surface properties.

Administration of MSCs into the arterial supply of the liver was examined in this study as an alternative route of intravenous injection to bypass lung entrapment. The inter-route extrapolation of this PBK model suggests that accumulation of MSCs in the liver significantly differs after intravenous and intra-hepatic arterial injection. Our PBK model confirmed that direct delivery of MSCs to the target organs may improve the therapeutic efficacy by increasing the accumulation of surviving cells in those organs. Compared to intravenous injection, transplantation via hepatic artery or portal vein could increase the amount of MSCs in the liver at 24 hours post-injection by 4-fold in humans (Fig. 6A), in agreement with the therapeutic efficacy study of the MSCs treatment of fulminant hepatic failure in pigs^29^. Our PBK model also allows the scale-up from the mice data to humans. In many studies on cell therapy, an accurate concentration of cells in organs is not available from humans^30^. One should be cautious in direct translation of the distribution results from mice to humans due to different concentration-time profiles of MSCs in organs between species. This PBK model could provide more accurate prediction by scaling up the profile from mice to human.

There is substantial evidence that administered MSCs would accumulate within sites of disease or injury. Local changes in microvessels and organ-derived attractants have been reported to affect the arrest and entrapment of MSCs in diseased organs^17^, while the time profiles of cell concentrations in unaffected organs may only slightly decrease^21^. Thus, parameter values of diseased organs in the PBK model would be different from normal organs. In the present model, MSC-specific parameters of diseased organs for myocardial infarction and liver cirrhosis were optimized separately from respective datasets, because these parameters were considered to be the most influential in the *in vivo* distribution of MSCs in disease, while other less influential parameters were kept the same. It would be of importance to investigate the MSC-specific parameters of target organs for each main type of diseases in the future.

In summary, we present the first model for characterizing and predicting the *in vivo* distribution of administered MSCs. Key ingredients in the model are species-specific physiological parameters (body weight, organ volume, blood volume and blood flow) and cell-specific parameters (partition coefficient, arrest rate, release rate and depletion rate). This model has been validated with multiple external datasets under widely different conditions and in different species, indicating potential inter-route and inter-species predictive capability. Based on our analysis, possible strategies to improve efficiency of cell-based therapies include bypassing the initial lung entrapment with administration to the arterial supply of target organs and enhancing organ-specific capture by modulating cell surface properties. This PBK model can be extended to other types of circulating cells by adapting the cell-specific parameters, and provides a general framework for the study of the *in vivo* distribution of therapeutic cells to design treatment protocols.

### Experimental Procedures

#### Cell preparations

The mouse GFP-MSCs used in this study were kindly provided by Dr. Mike Doran (Queensland University of Technology). The MSCs were isolated, characterized and cultured from inbred C57BL/6 mice transgenic for GFP under the control of the ubiquitin promoter as previously described^31^,^32^. All experiments involving MSCs were performed at passage 8–12, tested negative for mycoplasma contamination, and <80% confluence. The average diameter of suspended MSCs was measured from fifty different cells from twelve representative image fields.

#### *In vivo* transplantation and imaging of MSCs

Male 20-week-old BALB/c nude mice were purchased from the Animal Resource Centre (Perth, Western Australia). All animal procedures were approved by the Animal Ethics Committee of the University of Queensland and were carried out in accordance with Australian Code for the Care and Use of Animals for Scientific Purposes 8th edition. Mice were anaesthetized initially by an intraperitoneally injection of ketamine hydrochloride (80 mg/kg) and xylazine (10 mg/kg). Body temperature was controlled by placing mice on a heating pad set to 37 °C. 150 μl of a suspension of 5 × 10^5^ MSCs was injected with a 27 gauge needle through the tail vein. Prior to injection, the MSCs were maintained at 4 °C, and the cells were gently resuspended with a pipette to ensure no aggregation before injection.

MPM was performed using the DermaInspect system (Jen-Lab GmbH, Jena, Germany) equipped with an ultrashort (85 fs pulse width) pulsed mode-locked 80-MHz titanium sapphire laser (MaiTai, Spectra Physics, Mount View, CA, USA). The excitation wavelength was set to 740 nm for organ autofluorescence and 900 nm for GFP signals, with emission signal ranges of 350 to 450 nm and 450 to 515 nm established respectively through the use of BG39 bandpass filters (BG39, Schott glass color filter, Schott MG, Mainz, Germany). Images were recorded with oil-immersion 40x objectives (Carl Zeiss, Germany). The laser power was set to 15 mW for 40x magnification imaging, and the acquisition time for obtaining the images was 7.4 seconds per frame. Intravital imaging of the mouse liver was performed as previously described^33^,^34^. Twenty-four images from twelve non-overlapping fields were collected per mouse (*n* = 5) without the use of randomization and blinding. Normal saline was used to keep the liver and other organs moist and attached to the cover glass throughout the experiment. Analysis and overlay of the fluorescence intensity images was done using ImageJ 1.44p (National Institutes of Health, USA). The diameter of cells was measured along three different arbitrary lines within a cell, and tested on eighteen different cells from six representative image fields.

Organ specimens from sites of MPM imaging were fixed in 4% buffered formalin and embedded in paraffin. Serial sections were obtained for Hematoxylin & Eosin (H&E) stain to evaluate histopathologic features. The OlyVIA software 2.6 (Olympus, Münster, Germany) was used to visualize and scan the slides.

#### Measurement of donor MSCs in recipient organs

Animals (*n* = 5) were sacrificed at designated times (5 min, 15 min, 1, 3, 10, and 20 hour post-injection). The blood and major organs were removed and weighed. Red blood cells were lysed and single-cell suspensions of organs were obtained as previously described^35^. The total number of GFP-MSCs in each single-cell suspension of organs was measured and analyzed by flow cytometry using a FACS Calibur (Accuri C6, BD, San Jose, CA, USA) as previously described^36^. As negative controls, single-cell suspensions of organs from naive mice were run in parallel. Light scattering parameters were set to exclude dead cells and debris.

#### Mathematical description of the model

The model structure was based on published PBK models simulating the distribution (using distribution coefficient) and uptake-release-excretion processes (using uptake, release and excretion rate constant) of inert nanoparticles^37^. The model assumes a fast process of MSCs transported into various levels of blood vessels in organs. The partition coefficient *P* is used to correlate the concentration of MSCs between the blood within the organ and the venous blood leaving the organ. Given that the microvascular environment varies between organs, the partition coefficient is assumed to be different between organs. The equation describing this correlation is:


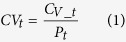


where *CV*_*t*_ (cell/L) is the concentration of MSCs in the venous blood leaving the organ *t*, *C*_*V_t*_ (cell/L) is the concentration of MSCs in the vascular space within the organ *t*, *P*_*t*_ (unitless) is the partition coefficient of the organ *t*.

Since a fraction of MSCs could be arrested in organs and isolated from blood circulation, these MSCs are described separately as in the extravascular space of organ. In the blood and organs, elimination of MSCs after depletion occurs as a clearance route from the body. The arrest-release-depletion approach of MSCs was described as a first-order process. The equations describing these processes are:

For vascular space


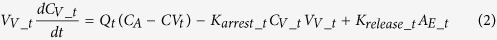


For the arrested MSCs as in the extravascular space


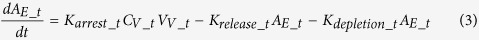


*V*_*V_t*_ (L) is the volume of blood vessels in the organ *t*, *Q*_*t*_ (L/h) is the blood flow to the organ *t*, *C*_*A*_ (cell/L) is the concentration of MSCs in the arterial blood, *A*_*E_t*_ (cell) is the amount of arrested MSCs and isolated from blood circulation as in the extravascular space of organ *t*, *K*_*arrest_t*_ (h^−1^) is the arrest rate constant of MSCs in the organ *t*, *K*_*release_t*_ (h^−1^) is the release rate constant of MSCs in the organ *t*, and *K*_*depletion_t*_ (h^−1^) is the depletion rate constant of MSCs in the organ *t*. Mass balance equations used in the model are presented in the Supplementary Note.

#### Implementation and parameterization of the model

The PBK model was implemented in Berkeley Madonna version 8.3.18 (Berkeley, CA, USA). Mass balance equations used in the model are presented in the supplementary. All physiological parameter values (body weight, organ volume, blood volume and blood flow) were from the literature and are given in Table S1. MSC-specific parameters (partition coefficient, arrest rate constant, release rate constant and depletion rate constant) were optimized by using both curve fitter in Berkeley Madonna automatically and a manual approach to obtain a visually reasonable fit to the experimental biodistribution data of GFP- MSCs intravenously injected into mice.

#### Sensitivity analyses

To determine the effect of the parameters on the model solution, sensitivity analysis was performed for the parameters in the target organs. The value of each parameter was increased by 0.1%, the model simulations were repeated, and the new MSCs concentrations noted. The relative sensitivity coefficients for significant parameters were calculated using the following equation:


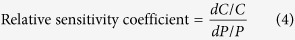


where *C* (cell/L) is the concentration of MSCs, and *P* is the parameter value. A positive RSC indicates a direct association between the model output and the corresponding parameter, while a negative RSC suggests the model output is inversely correlated with the specific parameter. The RSC values with absolute values higher than 0.5 are considered as highly sensitive.

#### Model evaluation with independent data

The predictive capability of our PBK model was evaluated with external datasets from different species^7^,^21^,^22^,^26^,^27^. To facilitate comparisons among the various studies, all concentrations were normalized to the number of MSCs per kg of organ. The physiological parameter values of rats and humans were obtained from the literature and are given in Table S1. MSC-specific parameters were assumed to be the same for mice, rats and humans. The overall goodness-of-fit between predicted and measured values was further analyzed with linear regression. To compare the predictive capability of model with different parameters, bias (mean prediction error [MPE]) and precision (mean absolute prediction error [MAPE]) are calculated with 95% confidence intervals (CIs) using the following equation:


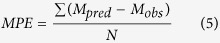



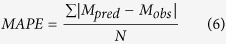


where *M*_*pred*_ is the predicted value, *M*_*obs*_ is the observed value, and *N* is number of time points. The statistical analysis was done using GraphPad Prism v 6.04 (GraphPad Software Inc., La Jolla, California).

## Additional Information

**How to cite this article**: Wang, H. *et al.* A physiologically based kinetic model for elucidating the *in vivo* distribution of administered mesenchymal stem cells. *Sci. Rep.* **6**, 22293; doi: 10.1038/srep22293 (2016).

## Supplementary Material

Supplementary Information

## Figures and Tables

**Table 1:** MSC-specific parameters used in the PBK model estimated by curve fitting.

Parameter (unit)	Description	Blood	Lung	Liver	Spleen	Kidney	Heart	Rest of body
*P* (unitless)	Partition coefficients	–	742.733	262.699	1633.24	305.351	3.097	6.765
*K*_*arrest*_ (h^−1^)	Arrest rate constant	–	5.434	1.395	0.608	1.727	1.251	0.143
*K*_*release*_ (h^−1^)	Release rate constant	–	0.108	0.066	0.856	0.054	0.016	0.957
*K*_*depletion*_ (h^−1^)	Depletion rate constant	0.636	0.0589	0.060	0.002	0.151	0.039	0.148
