# Supplementary material for: A physiologically based kinetic model for elucidating the in vivo distribution of administered mesenchymal stem cells
Source: Sci Rep. 2016 Feb 29;6:22293. doi: 10.1038/srep22293 (PMC4770280; doi:10.1038/srep22293)
Supplement: Supplementary Information [file srep22293-s1.pdf]

A physiologically based kinetic model for elucidating the *in vivo* distribution of administered mesenchymal stem cells

Haolu Wang, Xiaowen Liang, Zhi Ping Xu, Darrell H. G. Crawford, Xin Liu, Michael S. Roberts

Supplementary Figures

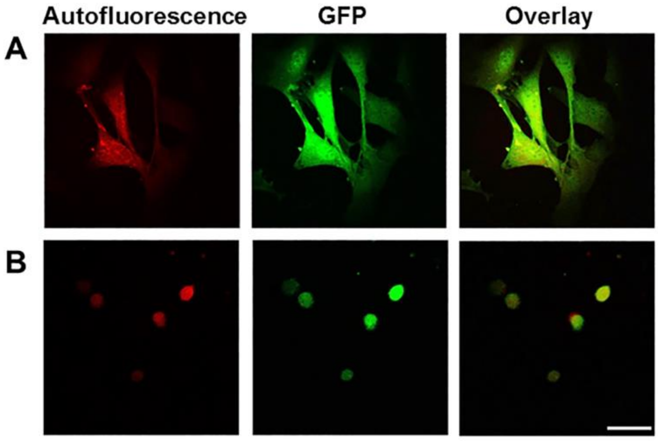

Figure S1 Morphology of MSCs *in vitro* imaged by MPM. (A) MSCs proliferated in the culture plate had a typical fibroblast-like morphology and were evenly distributed on the plate. (B) The suspended MSCs in mouse blood. Images were recorded at $\lambda_{Exc}/\lambda_{Em}$: 740/350 to 450 nm for the endogenous autofluorescence of MSCs (red, left column), and $\lambda_{Exc}/\lambda_{Em}$: 900/450 to 515 nm for fluorescence of GFP (green, middle column). The right column represents fused images. Scale bar: 40 μm.

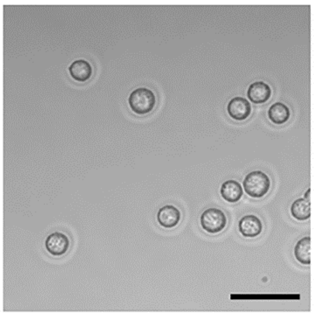

Figure S2 Morphology of MSCs *in vitro* imaged by bright-field microscopy. The MSCs were suspended in PBS. Scale bar: 40 µm.

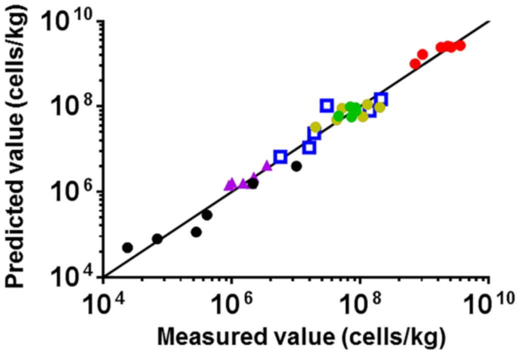

Figure S3 Goodness-of-fit plot of model calibration. Model predictions and experimental data were analyzed using linear regression. The linear regression coefficient (R^2) is 0.966 (n = 36).

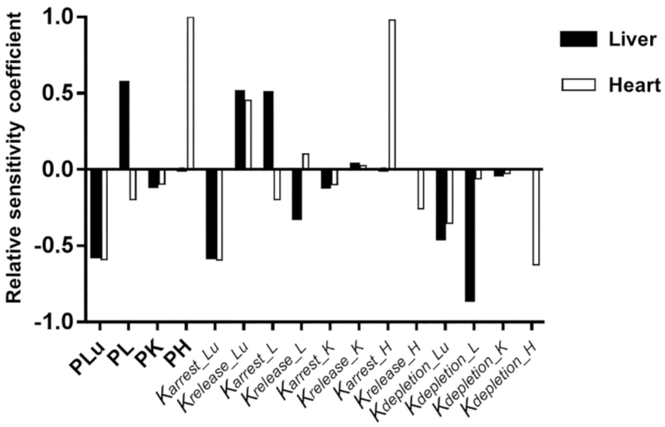

Figure S4 Sensitivity analyses for the MSC concentration in mouse liver and heart. Positive values indicate that MSC concentration increases when the parameter value increases, while

negative values indicate that MSC concentration decreases when the parameter value increases. P, partition coefficient; K_{arrest}, arrest rate constant; $K_{release}$, release rate constant; $K_{depletion}$, depletion rate constant; Lu, lung; L, liver; K, kidney; H, heart.

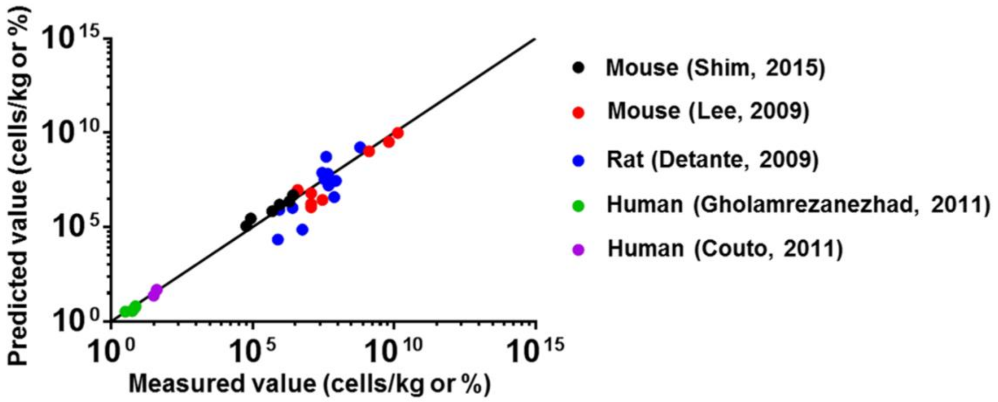

Figure S5 Goodness-of-fit plot of model evaluation. Model predictions and experimental data from independent external studies [1-5] were analyzed using linear regression. The linear regression coefficient (R^2) is 0.922 (n = 41).

Supplementary Tables

Table S1 Physiological parameters used in the PBK model

Parameter (unit)	Mouse	Rat	Human
Body weight (kg)	0.02	0.25	70
Cardiac output (L/hour/kg$^{0.75}$)	16.5	15	12.89
Blood flow to organ (fraction of cardiac output, unitless)			
Lung	1.00	1.00	1.00
Liver	0.161	0.25	0.227
Spleen	0.011	0.01125	0.01205
Kidney	0.091	0.141	0.175
Heart	0.035	0.035	0.037
Organ volumes (fraction of body weight, unitless)			
Lung	0.007	0.005	0.014
Liver	0.055	0.034	0.026
Spleen	0.005	0.0025	0.0026
Kidney	0.0017	0.007	0.00448
Heart	0.004	0.0022	0.0048
Blood	0.0085	0.074	0.079
Volume fraction of blood in organs (unitless)			
Lung	0.50	0.36	0.30
Liver	0.31	0.21	0.11
Spleen	0.17	0.22	0.51

Kidney	0.24	0.16	0.36
Heart	0.26	0.26	0.07
Rest of body	0.04	0.04	0.01

All values are from the literature [6-8]

Table S2 Disease-specific parameters of target organs estimated by curve fitting

Parameter (unit)	Description	Liver (Cirrhosis)	Heart (MI)
P (unitless)	Partition coefficient	376.074	2.311
K_{arrest} (h^{-1})	Arrest rate constant	5.793	6.823
$K_{release}$ (h^{-1})	Release rate constant	0.094	0.025
$K_{depletion}$ (h^{-1})	Depletion rate constant	0.098	0.029

MI: myocardial infarction.

Table S3 Predictive capability of the PBK model with original or disease-specific parameters

Variable	Disease	Parameter	Bias (MPE, SEM)	Precision (MAPE, SEM)
MSC concentration of heart	MI	Original	1.079×10^{7} (7.898×10^{6})	1.113×10^{7} (7.659×10^{6})
		Disease-specific	2.975×10^{6} (4.778×10^{6})	7.361×10^{6} (4.038×10^{5})
Proportion of MSCs in the liver	Cirrhosis	Original	-1.186 (0.3817)	1.242 (0.3343)
		Disease-specific	0.060 (0.3885)	0.690 (0.1812)

MI: myocardial infarction
MPE: mean prediction error
MAPE: mean absolute prediction error
SEM: standard error of the mean

Supplementary equations

Mass balance equations

For venous blood:

$$V_{Vb}\frac{dCV_{Vb}}{dt} = (Q_L CV_L + Q_S CV_L + Q_K CV_K + Q_H CV_H + Q_{Bo} CV_{Bo}) - Q_{Lu} CV_{Vb} - K_{depletion\_v} V_{Vb} CV_{Vb}$$

For arterial blood:

$$V_A\frac{dC_A}{dt} = Q_{Lu}(C_{V\_Lu} - C_A)$$

For lung:

$$CV_{Lu} = \frac{C_{V\_Lu}}{P_{Lu}}$$

For vascular space

$$V_{V\_Lu}\frac{dC_{V\_Lu}}{dt} = Q_{Lu}(CV_{Vb} - CV_{Lu}) - K_{arrest\_Lu}C_{V\_Lu}V_{V\_Lu} + K_{release\_Lu}A_{E\_Lu}$$

For the arrested MSCs as in the extravascular space

$$\frac{dA_{E\_Lu}}{dt} = K_{arrest\_Lu}C_{V\_Lu}V_{V\_Lu} - K_{release\_Lu}A_{E\_Lu} - K_{depletion\_Lu} \times A_{E\_Lu}$$

MSC concentration in the lung is given by:

$$C_{Total\_Lu} = \frac{C_{V\_Lu}V_{V\_Lu} + A_{E\_Lu}}{V_{Lu}}$$

For liver:

$$CV_{L} = \frac{C_{V\_L}}{P_{L}}$$

For vascular space

$$V_{V\_L}\frac{dC_{V\_L}}{dt} = Q_{L}C_{A} + Q_{S}C_{V\_S} - (Q_{L} + Q_{S})C_{V\_L} - K_{arrest\_L}C_{V\_L}V_{V\_L} + K_{release\_L}A_{E\_L}$$

For the arrested MSCs as in the extravascular space

$$\frac{dA_{E\_L}}{dt} = K_{arrest\_L}C_{V\_L}V_{V\_L} - K_{release\_L}A_{E\_L} - K_{depletion\_L} \times A_{E\_L}$$

MSC concentration in the liver is given by:

$$C_{Total\_L} = \frac{C_{V\_L}V_{V\_L} + A_{E\_L}}{V_{L}}$$

For spleen:

$$CV_{S} = \frac{C_{V\_S}}{P_{S}}$$

For vascular space

$$V_{V\_S}\frac{dC_{V\_S}}{dt} = Q_S(C_A - CV_S) - K_{arrest\_S}C_{V\_S}V_{V\_S} + K_{release\_S}A_{E\_S}$$

For the arrested MSCs as in the extravascular space

$$\frac{dA_{E\_S}}{dt} = K_{arrest\_S}C_{V\_S}V_{V\_S} - K_{release\_S}A_{E\_S} - K_{depletion\_S} \times A_{E\_S}$$

MSC concentration in the spleen is given by:

$$C_{Total\_S} = \frac{C_{V\_S}V_{V\_S} + A_{E\_S}}{V_S}$$

For kidney:

$$CV_K = \frac{C_{V\_K}}{P_K}$$

For vascular space

$$V_{V\_K}\frac{dC_{V\_K}}{dt} = Q_K(C_A - CV_K) - K_{arrest\_K}C_{V\_K}V_{V\_K} + K_{release\_K}A_{E\_K}$$

For the arrested MSCs as in the extravascular space

$$\frac{dA_{E\_K}}{dt} = K_{arrest\_K}C_{V\_K}V_{V\_K} - K_{release\_K}A_{E\_K} - K_{depletion\_K} \times A_{E\_K}$$

MSC concentration in the kidney is given by:

$$C_{Total\_K} = \frac{C_{V\_K}V_{V\_K} + A_{E\_K}}{V_K}$$

For heart:

$$CV_H = \frac{C_{V\_H}}{P_H}$$

For vascular space

$$V_{V\_H}\frac{dC_{V\_H}}{dt} = Q_H(C_A - CV_H) - K_{arrest\_H}C_{V\_H}V_{V\_H} + K_{release\_H}A_{E\_H}$$

For the arrested MSCs as in the extravascular space

$$\frac{dA_{E\_H}}{dt} = K_{arrest\_H} C_{V\_H} V_{V\_H} - K_{release\_H} A_{E\_H} - K_{depletion\_H} \times A_{E\_H}$$

MSC concentration in the heart is given by:

$$C_{Total\_H} = \frac{C_{V\_H} V_{V\_H} + A_{E\_H}}{V_H}$$

For the rest of the body:

$$CV_{Bo} = \frac{C_{V\_Bo}}{P_{Bo}}$$

For vascular space

$$V_{V\_Bo} \frac{dC_{V\_Bo}}{dt} = Q_{Bo}(C_A - CV_{Bo}) - K_{arrest\_Bo} C_{V\_Bo} V_{V\_Bo} + K_{release\_Bo} A_{E\_Bo}$$

For the arrested MSCs as in the extravascular space

$$\frac{dA_{E\_Bo}}{dt} = K_{arrest\_Bo} C_{V\_Bo} V_{V\_Bo} - K_{release\_Bo} A_{E\_Bo} - K_{depletion\_Bo} \times A_{E\_Bo}$$

MSC concentration in the rest of body is given by:

$$C_{Total\_Bo} = \frac{C_{V\_Bo} V_{V\_Bo} + A_{E\_Bo}}{V_{Bo}}$$

Nomenclature (units)

A_E: Amount of arrested MSCs as in the extravascular space of each compartment (cell)

C_{Total}: Average MSC concentration of each compartment (cell/kg)

C_V: MSC concentration in the vascular space of each compartment (cell/kg)

CV: MSC concentration in the venous blood (cell/kg)

K_{arrest}: Arrest rate constant of MSCs (h^{-1})

$K_{release}$: Release rate constant of MSCs (h^{-1})

$K_{depletion}$: Depletion rate constant of MSCs in the organ (h^{-1})

P: Partition coefficient (unitless)

Q: Blood flow to each organ (L/h)

V: Total volume of each compartment (L)

V_V: Volume of vascular space of each compartment (L)

Subscripts

Vb: Venous blood

A: Arterial blood

Lu: Lung

L: Liver

S: Spleen

K: Kidney

H: Heart

Bo: The rest of body

Supplementary References

1. Shim, G. *et al.* Pharmacokinetics and in vivo fate of intra-articularly transplanted human bone marrow-derived clonal mesenchymal stem cells. *Stem Cells Dev* **24**, 1124-1132 (2015).
2. Lee, R. H. *et al.* Intravenous hMSCs improve myocardial infarction in mice because cells embolized in lung are activated to secrete the anti-inflammatory protein TSG-6. *Cell Stem Cell* **5**, 54-63 (2009).
3. Detante, O. *et al.* Intravenous administration of 99mTc-HMPAO-labeled human mesenchymal stem cells after stroke: in vivo imaging and biodistribution. *Cell Transplant* **18**, 1369-1379 (2009).
4. Gholamrezanezhad, A. *et al.* In vivo tracking of 111In-oxine labeled mesenchymal stem cells following infusion in patients with advanced cirrhosis. *Nucl Med Biol* **38**, 961-967 (2011).
5. Couto, B. G. *et al.* Bone marrow mononuclear cell therapy for patients with cirrhosis: a Phase 1 study. *Liver Int* **31**, 391-400 (2011).
6. Brown, R. P., Delp, M. D., Lindstedt, S. L., Rhomberg, L. R. & Beliles, R. P. Physiological parameter values for physiologically based pharmacokinetic models. *Toxicol Ind Health* **13**, 407-484 (1997).
7. Sterner, T. R., Ruark, C. D., Covington, T. R., Yu, K. O. & Gearhart, J. M. A physiologically based pharmacokinetic model for the oxime TMB-4: simulation of rodent and human data. *Arch Toxicol* **87**, 661-680 (2013).
8. Lin, Z., Monteiro-Riviere, N. A. & Riviere, J. E. A physiologically based pharmacokinetic model for polyethylene glycol-coated gold nanoparticles of different sizes in adult mice. *Nanotoxicology*, epub ahead of print (2015).
